# Model-Based Angular Scan Error Correction of an Electrothermally-Actuated MEMS Mirror

**DOI:** 10.3390/s151229840

**Published:** 2015-12-10

**Authors:** Hao Zhang, Dacheng Xu, Xiaoyang Zhang, Qiao Chen, Huikai Xie, Suiqiong Li

**Affiliations:** 1 School of Electronic and Information Engineering, Soochow University, Suzhou 215006, China; zhanghao8.sz@jsoa.net (H.Z.); lisuiqiong@suda.edu.cn (S.L.); 2 Department of Electrical and Computer Engineering, University of Florida, Gainesville, Florida, FL 32611-6200, USA; xzhang292@ufl.edu (X.Z.); hkxie@ece.ufl.edu (H.X.); 3 Wuxi WiO Technology Co. Ltd, Wuxi 214000, China; qchen@wiotek.com

**Keywords:** electrothermal actuator, MEMS mirror, static deviation, model based open-loop control

## Abstract

In this paper, the actuation behavior of a two-axis electrothermal MEMS (Microelectromechanical Systems) mirror typically used in miniature optical scanning probes and optical switches is investigated. The MEMS mirror consists of four thermal bimorph actuators symmetrically located at the four sides of a central mirror plate. Experiments show that an actuation characteristics difference of as much as 4.0% exists among the four actuators due to process variations, which leads to an average angular scan error of 0.03°. A mathematical model between the actuator input voltage and the mirror-plate position has been developed to predict the actuation behavior of the mirror. It is a four-input, four-output model that takes into account the thermal-mechanical coupling and the differences among the four actuators; the vertical positions of the ends of the four actuators are also monitored. Based on this model, an open-loop control method is established to achieve accurate angular scanning. This model-based open loop control has been experimentally verified and is useful for the accurate control of the mirror. With this control method, the precise actuation of the mirror solely depends on the model prediction and does not need the real-time mirror position monitoring and feedback, greatly simplifying the MEMS control system.

## 1. Introduction

MEMS mirrors have been crucial parts in various optical devices and systems, such as scanning mirrors for target detection and measurement, optical switches for telecommunication, and scanning engines for optical endoscopy [1]. MEMS mirror based optical switches aim and align the optical beam to the designed paths with extremely high accuracy and fast speed [2]. MEMS technique allows the fabrication of optical switch arrays containing a large amount of micro-mirrors, which is the key technique for optical cross-connects (OXC), spatial light modulation, portable displays, and adaptive optics systems [3]. The capability of precisely controlling the movement and the position of micro-mirrors would determine the performance of MEMS-based optical systems. Therefore, developing advanced methods to improve the degree of the accuracy of mirror actuation has attracted great attentions.

Actuation mechanisms that have been used to drive MEMS mirrors include electrostatic [4], electrothermal [5,6], electromagnetic [2], and piezoelectric [7]. Generally, electrostatic force has been the most widely used in the actuators of MEMS mirrors due to several advantages, such as high speed, low power consumption, good reliability and controllability [8]. However, electrostatic actuators require high driving voltage and larger footprint because of the comb-structure driving electrodes, limiting their applications in certain fields such as endoscopic imaging. On the other hand, electrothermal actuators can generate large displacement and high force output at low voltage and achieve relatively smaller size and lighter weight [9,10], thus they are preferred in optical probes used in *in vitro* diagnostic medical devices, where large scan angle at low drive voltage is required for safe use inside human body [11]. A major drawback of conventional thermal activated bimorph MEMS mirrors is the large initial tilt angle. In order to overcome this problem, an inverted-series-connected (ISC) bimorph actuator MEMS mirror was recently designed and fabricated to achieve zero initial tilt angle [12]. The S-shaped ISC bimorph actuator proposed by Todd [13] overcomes both mirror plate shift and rotation-axis shift problems. High resolution 3D imaging has been achieved using the miniature optical coherence tomography (OCT) probe equipped with micromirrors driven by the S-shaped ISC bimorph actuators [14]. In this ISC MEMS design, each mirror is actuated by four independently-controllable actuators. Even through by design the four actuators would provide symmetric actuation, the imperfections of microfabrication processes unavoidably cause resistance, structural and mechanical property variations among the four actuators, resulting in actuation characteristics non-uniformity and nonlinear voltage–angle relationship. This will bring inaccurate mirror movement and cause scanning deformation, thus obstruct the applications of the MEMS mirror in imaging, displays and sensing. The elimination of the static deviation stemming from the fabrication process and the dynamic vibration is essential for this ISC MEMS mirror to be used in high performance optical systems. The common method to compensate the actuation error of MEMS mirrors is to first establish proper models for micromirror actuation and then build a closed-loop control system with position or angular sensors feeding back the mirror gesture data [15,16,17,18,19]. However, this method needs accurate real-time motion data of the mirror from sensors and sophisticated algorithms to promptly correct the deviation. In this way, it greatly increases the package size and cost.

In this work, we developed a mathematical mode based control system for actuating two-axis ISC MEMS mirror, which does not require the position or angular sensors to provide feedback data. A simple model that can accurately predict the actuation characteristics of the ISC MEMS mirror has been established. Based on this model, an open-loop control system was built to calculate the desired actuation signals. Actuating with this model-based open loop control system can effectively correct and compensate the non-uniformity of the driving force and the nonlinearity between the input voltage and the mirror-plate position. The experimental results showed that the scan error of the ISC MEMS mirror was greatly reduced by applying the open-loop control system, indicating the validity of this model-based compensation method.

## 2. Experimental Section

### 2.1. Design and Fabrication of Two-Axis ISC MEMS Mirror

**Figure 1:**
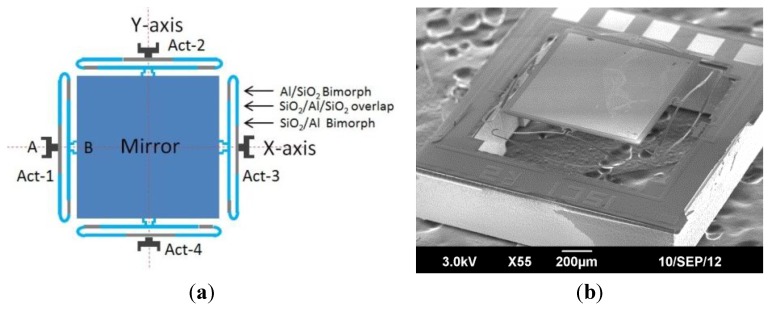
Two-axis ISC MEMS mirror. (**a**) Device design: there are four ISC actuators whose A ends are anchored on the substrate and B ends connect to the central mirror plate; (**b**) SEM of a fabricated device: the initial elevation of the mirror plate is 240 μm, the mirror plate is 1.0 mm × 1.0 mm, and the whole device size is 1.5 mm × 1.5 mm.

**Figure 2:**
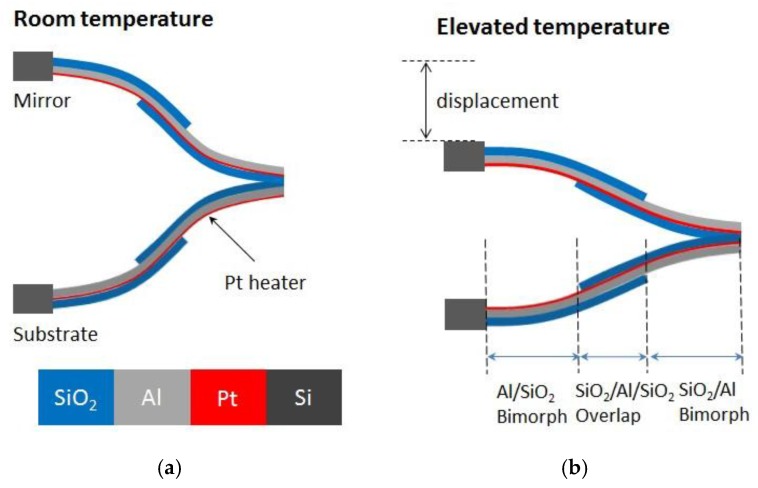
Side view of the ISC S-shaped bimorph actuator. (**a**) No voltage is applied; (**b**) The end of the ISC actuator pulls down the mirror plate when a voltage is applied.

As shown in Figure 1, the two-axis MEMS mirror used in this study is based on electro-thermal ISC actuation mechanism, where each mirror unit is comprised of a mirror plate that is supported and actuated by four pairs of ISC bimorph actuators. The mirror plate consists of an aluminum mirror surface and a silicon layer underneath [13], and is supported by the four actuators symmetrically attached to each side of the mirror. One end of each actuator is anchored on the silicon substrate and the other end connects to the mirror plate, as illustrated in Figure 1a. The scanning electron microscope (SEM) image of a fabricated MEMS mirror is shown in Figure 1b. The four actuators can be controlled separately to perform either piston or tip-tilt motion. The structure of the ISC actuator is shown in Figure 2. This mirror is designed for OCT probe, where a relatively large mirror is more suitable [11]. Conventionally, an electrothermal actuator is a bimorph that consists of two materials with different thermal expansion coefficients. When the bimorph experiences a temperature change, a large vertical displacement generates at the tip, providing required driving force. However, there exists a tangential tip angle as well as a tip lateral shift for single bimorph actuation. An inverse-series-connected (ISC) bimorph structure formed by connecting two inverse bimorphs can compensate the tangential angle in its tip displacement, but still leave non zero lateral shift. In this study, the actuator was fabricated by connecting two ISC beams in folded fashion, as shown in Figure 2. Each actuator is comprised of two identical and symmetric ISC bimorphs, and each ISC bimorph has three segments: Al/SiO_2_ inverted bimorph segment, SiO_2_/Al/SiO_2_ overlap, and SiO_2_/Al non-inverted bimorph segment [20]. A Pt layer is embedded along the ISC bimorph actuators as heating resistors. When a voltage is applied, the heat generated by the Pt resistors will change the bimorph temperature. Due to the different coefficients of thermal expansion of Al and SiO_2_, the bimorph will bend, causing the bending of the Al/SiO2 bimorph. Due to the structure of two symmetric ISC bimorph, the tangential tile angle and lateral shift are cancelled and displacements are added up. Thus, pure large vertical displacement can be achieved, as shown in Figure 2b. With four symmetric ISC actuators controlling the four sides of the mirror plate, the mirror plate can move vertically and generate angular scan in two axes. The device is fabricated using a combined bulk- and surface-micromachining process and SOI (Silicon On Insulator) wafers are selected to ensure the flatness of the mirror plate. The process includes Pt heater lift-off, SiO_2_ PECVD, Al sputter deposition, SiO_2_ RIE, and silicon DRIE, as described in [6].

Theoretically, the four independent and symmetric ISC bimorph actuators described above can provide uniform and precise control of mirror’s piston and tilting motion. However, the fabrication imperfection and mechanical coupling between actuators will cause errors in output angle tilt and piston displacement. Therefore, we developed a simple model to predict the output signal based on numerous measurements. Meanwhile, based on this model, an open-loop control system was built to achieve the desired actuation signals.

### 2.2. Quasi-Static Tip-Tilt-Piston Characteristics of the ISC MEMS Mirror

In order to establish the mathematical model that can predict the output signal of the actuators, the static response of the ISC bimorph actuators should first be characterized. The quasi-static tip-tilt-piston characteristics of the ISC actuation system was obtained by measuring the tilt angle and the height of the mirror plate when applying different driving signals.

#### 2.2.1. Angular Scan Measurements

**Figure 3:**
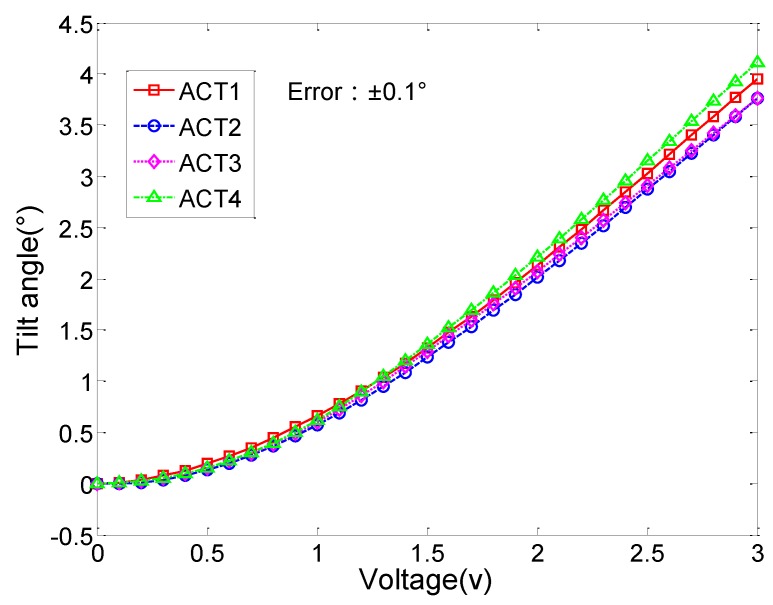
Tilt angle *vs.* voltage curve. The actuators were driven one by one.

The tilt angle of the mirror plate was measured with one actuator activated each time. The measurement data were plotted in Figure 3. About 4° scan angle (mechanical) was achieved at merely 3 V and the response curves are highly nonlinear at low actuation voltage. Figure 3 also shows that the response curves of the four actuators do not coincide and the deviation becomes larger with the increase of the actuation voltage. These differences and nonlinearity will affect the accuracy of the mirror actuation and lead to image distortion if applied for image scanning.

#### 2.2.2. Measurement of the Heights of the Mirror Plate at the Four Actuator Anchor Points

**Figure 4:**
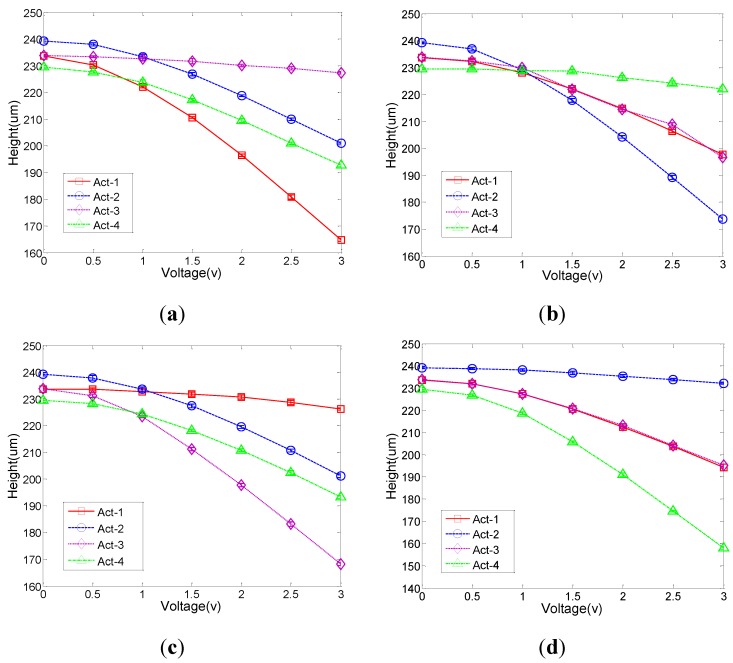
Height *versus* applied voltage. The voltage was applied to only one actuator. The heights of all four actuators were measured: (**a**) Only Actuator (ACT) 1 is active; (**b**) Actuator (ACT) 2 only; (**c**) Actuator (ACT) 3 only; and (**d**) Actuator (ACT) 4 only.

In order to evaluate the pointing accuracy of this 2-axis MEMS mirror, the heights (relative to the substrate surface) of the four actuator anchor points on the mirror plate were measured. Figure 4 plots the heights of the four anchor points when only one of the four actuators was activated. The experimental data show that when a driven voltage is applied to one actuator, the heights of all other actuators will be changed. Thus, there is thermal coupling and maybe mechanical coupling as well among the actuators. In addition, under the same drive voltage, different actuators will result in different height changes. This indicates there are electrical and mechanical variations among the four actuators.

#### 2.2.3. Identification of the Source of Actuation Errors

From the experimental data shown in Figure 3 and Figure 4, three main issues that cause actuation errors are identified, including the actuator non-uniformity, mechanical coupling, and initial elevation variation. These issues will lead to problems in pointing accuracy, scan pattern stability, repeatability, and scan center offset.

(a) Actuator non-uniformity

The differences of the four actuators mainly come from fabrication imperfections, for instance, thin-film layer thickness variation, non-uniform distribution of thermal stresses, and heater resistance variations. The powers generated by the Pt heaters will be different even with the same driving voltage due to the resistance difference. Even the same power will not generate the same displacement due to the stress and layer thickness.

(b) Mechanical coupling

**Figure 5:**
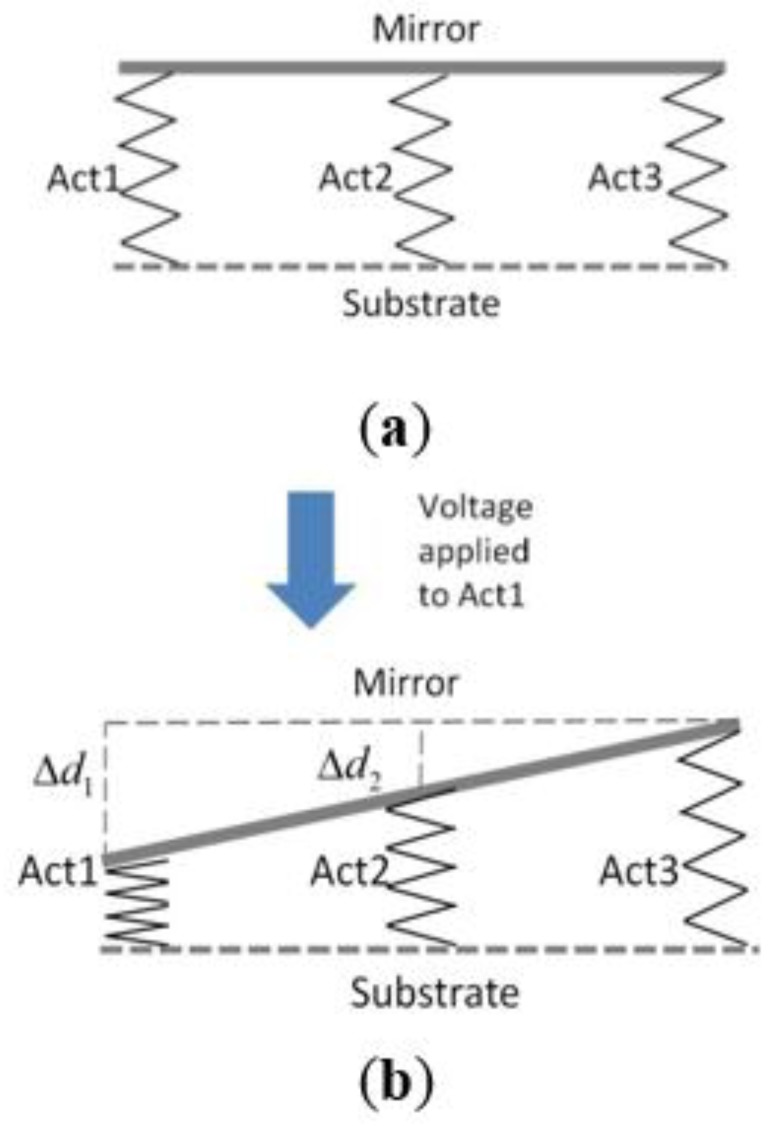
Cross-sectional view of the micro-mirror structure. ACT4 is placed behind ACT2: (**a**) no actuation and (**b**) voltage applied to ACT1 only. Act1 deforms by heat and produces height change Δ*d*_1_. ACT2 and ACT4 will also have height change Δ*d*_2_ pulled by the mirror plate.

Since the mirror plate is a rigid-body, the height change of one actuator will affect the heights of other actuators. Figure 5 illustrates the mechanical coupling among the actuators, where each actuator is modeled as a spring and the mirror plate as a rigid bar. When a voltage applied to ACT1 leads to a height change Δd1 by Joule heating, ACT2 and ACT4 will have a height change Δd2 pulled by the mirror plate. The two height changes are simply related as

Δ*d*_1_ = 2 × Δ*d*_2_ (1)

There is also a coupling between the active actuator and the other three inactive actuators. From the testing data, it is found when one actuator is driven, the opposite actuator is not stationary and all three inactive actuators have vertical displacement.

(c) Initial heights of the four anchor points on the mirror plate

From Figure 4, it can also be seen that the initial heights (*i.e.*, at zero voltage) of the side centers of the mirror plate are not completely uniform and the maximum deviation is 9.7 μm. Thus, the mirror plate has an initial tilt due to the fabrication imperfection and will be tilted even with the same voltage applied to all four actuators.

### 2.3. System Identification and Modeling

#### 2.3.1. Establish of Mathematical Model for Predicting Static Output of Actuation

A mathematical mode of the static response of the mirror driven by the four ISC actuators was established based on the experimental data of the height change of the mirror plate over the input voltages. Because this mirror is actuated by four input voltage signals and the mirror position is determined by the height of four actuators, it can be simplified to a four-inputting-four-outputting model. As presented in the previous section, actuation of any actuator causes the height changes of others. As shown in Figure 2, the height represents the displacement from the connection joint to the substrate surface. A 4 × 4 characteristic matrix is proposed, which relates the four actuation voltages as the input and the heights of the mirror plate at the four actuator anchor points as the output. The matrix equation is shown in Equation (2):

(2) $$\left(\begin{array}{cccc} f_{11} & f_{12} & f_{13} & f_{14}\\ f_{21} & f_{22} & f_{23} & f_{24}\\ f_{31} & f_{32} & f_{33} & f_{34}\\ f_{41} & f_{42} & f_{43} & f_{44} \end{array}\right)\left(\begin{array}{c} U_{1}\\ U_{2}\\ U_{3}\\ U_{4} \end{array}\right)=\left(\begin{array}{c} d_{1}\\ d_{2}\\ d_{3}\\ d_{4} \end{array}\right)$$

where U*_i_* is the input voltage to ACT-*i*, d*_i_* is the corresponding height of ACT-*i*, *f_i,j_* represents the coupling from ACT-*j* to ACT-*i*, and *i* or *j* = 1,2,3,4.

The identification is performed in the following procedure. The voltage ranging from 0 to 3 V is applied to one actuator, and all four actuators’ heights relative to the substrate surface are measured by an auto-focus microscope (OLYMPUS STM6, precision 0.1 μm). This measurement is done one by one from ACT1 to ACT4, and sixteen sets of height-voltage data are obtained. Then, these data are fitted using polynomial functions. The result shows that cubic functions can generate accurate fit of the height–voltage curves. Equation (3) gives the polynomial fit result for the case where only ACT1 is active.

(3) $$\begin{array}{l} d_{1}=1.378{u_{1}}^{3}-11.257{u_{1}}^{2}-1.568u_{1}+233.6\\ d_{2}=0.978{u_{1}}^{3}-7.391{u_{1}}^{2}+0.632u_{1}+239.3\\ d_{3}=0.067{u_{1}}^{3}-0.733{u_{1}}^{2}-0.560u_{1}+233.8\\ d_{4}=0.911{u_{1}}^{3}-6.848{u_{1}}^{2}+0.091u_{1}+229.4 \end{array}$$

where u_1_ is the voltage applied to Act1. The constant terms are the initial heights of the actuators. The four equations represent the position relations among the actuators when Act 1 is driven. For any given voltage applied to ACT1, the heights of all four actuators can be calculated using Equation (3).

In the same way, the other three sets of polynomial fitted equations can be obtained. Linear superposition of all four sets of equations yields a complete heights-voltage matrix as follows:

(4) $$A\times U^{3}+B\times U^{2}+C\times U+D=d$$

where $A=\left[\begin{array}{cccc} 1.378 & 0.711 & -0.089 & 0.867\\ 0.978 & 1.600 & 0.733 & 0.178\\ 0.067 & 0.422 & 1.822 & 0.778\\ 0.911 & 0.111 & 0.689 & 1.867 \end{array}\right]B=\left[\begin{array}{cccc} -=8679+ & -6.067 & -0.491 & -6.852\\ -7.391 & -12.290 & -6.486 & -1.343\\ -0.733 & -5.138 & -12.871 & -12.87\\ -12.87 & -1.538 & -6.191 & -6.1911 \end{array}\right]$ $C=\left[\begin{array}{cccc} -=.191 & -0.173 & -0.209 & -0.338\\ 0.632 & 0.636 & 0.188 & 0.103\\ -0.560 & -0.589 & 0.361 & -.3619\\ 0.091 & 1.141 & 0.280 & 0.955 \end{array}\right]d=\left[\begin{array}{c} 233.6\\ 239.2\\ 233.8\\ 229.5 \end{array}\right]U=\left[\begin{array}{c} u_{1}\\ u_{2}\\ u_{3}\\ u_{4} \end{array}\right]d=\left[\begin{array}{c} d_{1}\\ d_{2}\\ d_{3}\\ d_{4} \end{array}\right]$ U is a 4 × 1 voltage matrix and *u_i_* is the actuation voltage to ACT-*i*, D is the initial height matrix, d is a 4 × 1 matrix representing the final heights of the four actuators, and *d_i_* is the height of ACT-i. After the heights at different voltages are calculated with this model, the tilt angle of the mirror plate can be express as

(5) $$\theta_{x}=\arcsin\frac{\left|d_{1}-d_{3}\right|}{Wx}\theta_{y}=\arcsin\frac{\left|d_{2}-d_{4}\right|}{Wy}$$

where $\theta_{x}$ and $\theta_{y}$ are the angles of the mirror plate in X- and Y-axes, respectively, and *Wx* and *Wy* are the side lengths of the mirror plate in x and y, respectively.

**Figure 6:**
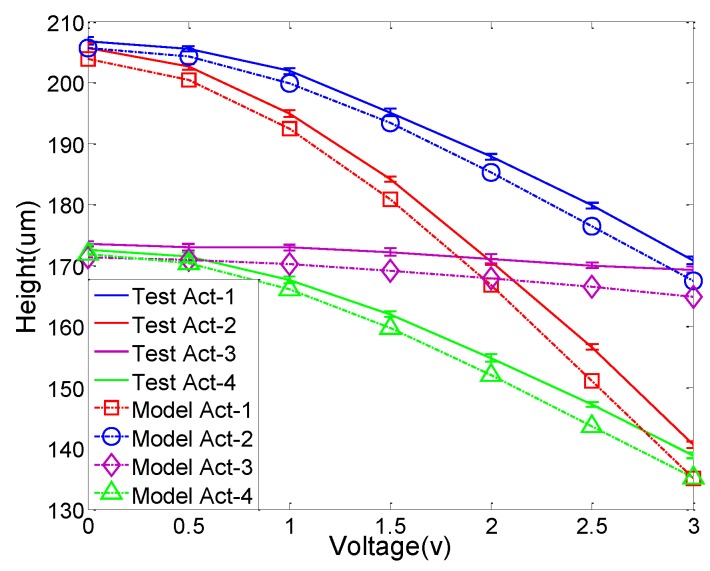
Plot of the experimental data and model prediction when the varying voltage is applied only to ACT1 and the voltages on ACT2, 3 and 4 are fixed at 1 V, 2 V and 2 V respectively.

**Figure 7:**
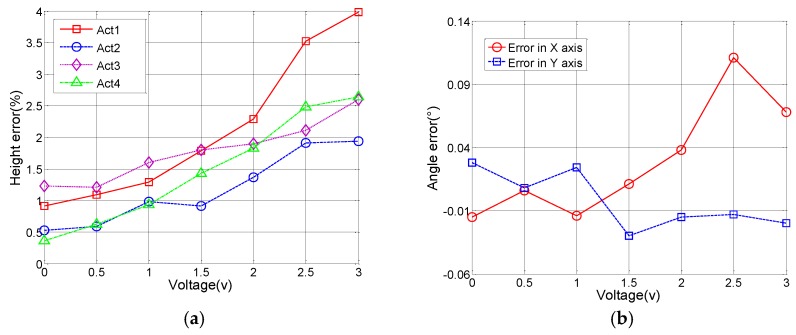
(**a**) The height difference between the model prediction and experimental data (from Figure 6); (**b**) The corresponding tilt angle error of the mirror plate.

To validate the accuracy and reliability of the model, different voltage combinations are applied to the actuators simultaneously. Figure 6 shows the comparison of the test data and model prediction in different conditions. In this case, the voltage applied to ACT1 is 0~3 V, and the voltages applied to ACT2, ACT3 and ACT4 are 1 V, 2 V and 2 V, respectively. Figure 7a plots the height difference between the experimental data and model prediction, where the average height difference is 1.64% with the maximum of 4.0%. The corresponding tilt angle error can be calculated using Equation (5). As shown in Figure 7b, the average angle error is 0.03°, with the maximum of 0.11° (out of 4°). Thus, the developed static model can predict the actuation response of the MEMS mirror within 4.0% error.

To keep the mirror tilt around the center point, an initial bias voltage is applied to all actuators and differential driving voltages are applied to both X- and Y-axes. The input angles of two axes can be converted to the heights for each actuator by the following equation:

(6) $$h_{1,3}=\frac{W\times \tan\theta_{x}}{2}\mp H_{b},h_{2,4}=\frac{W\times \tan\theta_{y}}{2}\mp H_{b}$$

where $\theta_{x}$ and $\theta_{y}$ are the angles of the mirror in X- and Y-axes, respectively, *h_i_* is the height of ACT-*i*, *W* is the width of mirror plate, and H_b_ is the initial height corresponding to the bias voltage.

#### 2.3.2. Construction of Compensated Control System Based on the Mathematical Model

**Figure 8:**
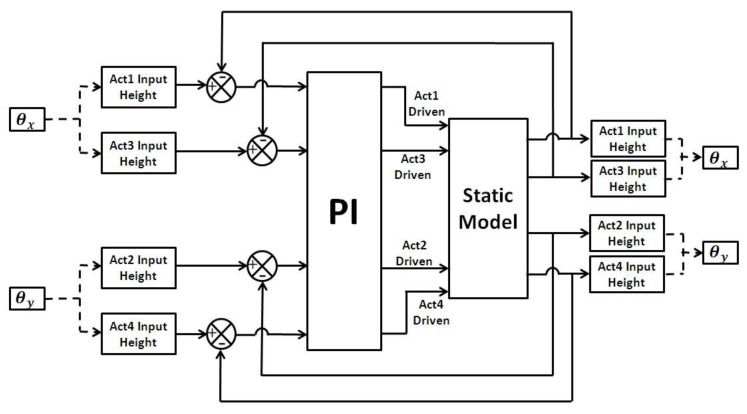
The block diagram of the control system with four inputs and outputs.

It would be ideal if the heights of all four actuators can be monitored and controlled in real time, but this would require integrated displacement sensors, which are difficult to integrate with the MEMS mirror. Fortunately, with the static model constructed in the previous section, the heights of all four actuators of the MEMS mirror can be obtained by inversing Equation (4). However, this model is a four-input, four-output system, which requires extensive computing resources and may have multiple sets of solutions. To solve this problem, we propose a proportional-integration (PI) algorithm based on the static model to generate the actuation voltage signals for open-loop control. The control system is shown in Figure 8. It includes a static model of the MEMS mirror and a PI feedback control.

As shown in Figure 8, any desired scan pattern, defined by a set of $\theta_{x}$ and $\theta_{y}$, is first converted to the four actuator heights, according to Equation (6), as the input signals of the PI controller. The PI controller generates a set of actuation signals as the input of the device static model that computes the actuator heights. The model-generated heights are then fed back to the input of the PI controller and compared with the desired heights. The feedback loop makes the difference between the desired and model-generated heights quickly approach zero, so the output voltage signals from the PI controller can be used to drive the actual MEMS mirror and achieve the desired scan pattern. In practice, the PI controller output signals are recorded and the recorded data are output to a digital-to-analog converter. The signals are further conditioned by a power amplifier before applied to the MEMS device.

Ziegler–Nichols method is used to calculate the PI parameters, *K_i_* and *K_p_*. *K_i_* is first set to zero, and then the proportional gain is increased until it reaches the ultimate gain, at which the output of the loop starts to oscillate [21]. Then, the PI parameters are set as the following:

(7) $$K_{p}=0.45K_{u},K_{i}=1.2K_{p}/P_{u}$$

where $K_{u}$ is the ultimate gain and $P_{u}$ is the oscillation period. For most of the 2-axis MEMS mirrors tested in this study, *K_u_* = 0.2 and *P_u_* = 375. The model achieves stable output in about 10 ms using these parameters. This system has no converging problems for the maximum heights up to 240 μm. The performance of this control system was verified and the results are discussed in Section 3.

### 2.4. Validation of the Static Model-Based Open-Loop Control System

**Figure 9:**
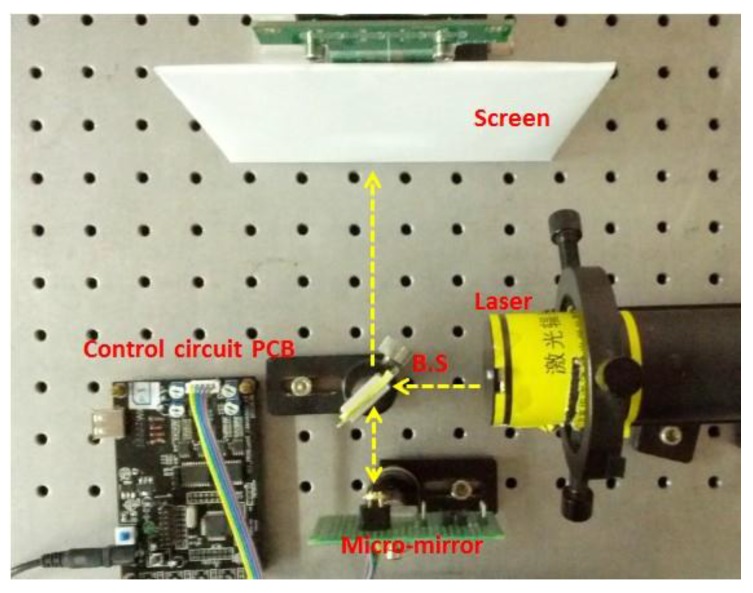
The laser scanning system that was constructed based on the MEMS mirror. The scanning can be driven by standard driven signals or by the model-based open-loop control system.

In order to validate the effectiveness of static model-based open-loop control, a laser scanning probe was constructed based on MEMS mirror described in Section 2.1. The MEMS mirror was driven by a control circuit that can provide standard triangle signals or signals compensated using the model-based open-loop control described in Section 2.3. The experimental setup is shown in Figure 9. The laser beam is directed to the center of the MEMS mirror by a beam splitter. Then the laser beam is reflected by the MEMS mirror, passes through the beam splitter and reaches the screen. Driving voltage signals are applied to the MEMS mirror to generate various scan patterns on the screen. The results that were generated using standard driven signals and compensated driven signals were compared to investigate the effectiveness of the developed control system.

## 3. Results and Discussion

### 3.1. Output Signal Simulation of the Compensated Control System 

**Figure 10:**
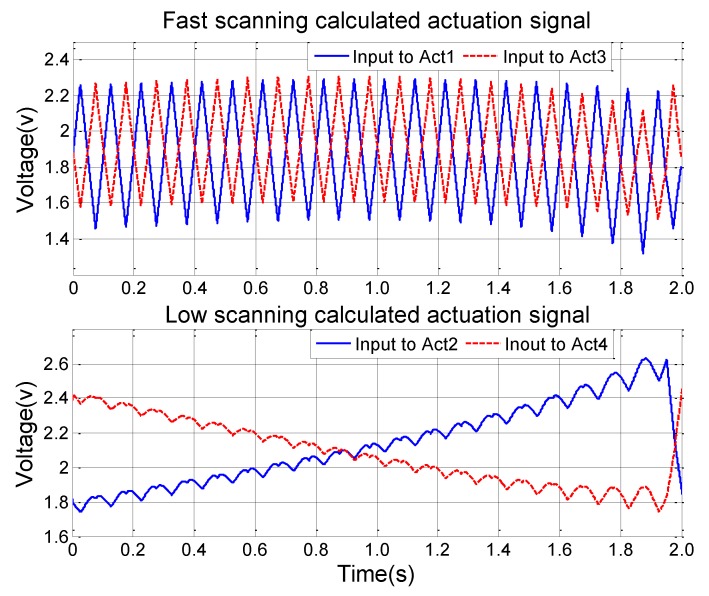
The calculated control signal including fast scan in X-axis and slow scan in Y-axis.

**Figure 11:**
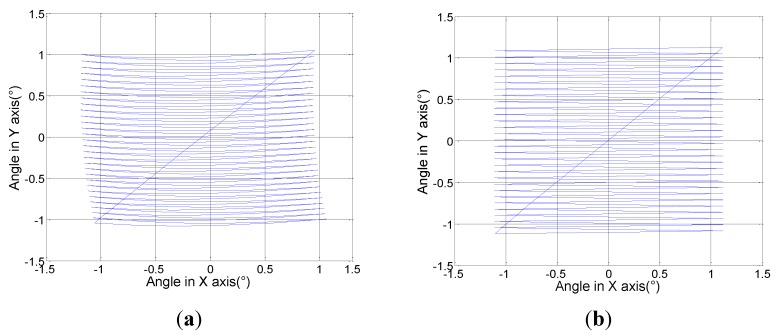
The comparison of controlled and non-controlled raster scan patterns. (**a**) Scan pattern with standard triangular voltage signals; (**b**) Scan pattern with the model-generated signals as in Figure 9.

To verify the performance of this control, a raster scan pattern is applied into the PI control model as the reference signal, which includes a fast scan signal and a slow scan signal. Both signals are triangular. After going through the PI feedback control, the actuation signals of all four actuators are obtained, as shown in Figure 10. The fast and low scan frequency ratio is 30:1. There is an amplitude change in the fast scan signal, which compensates the differences of the actuators. Jitters are also present in the slow scan signal, which can compensate the influence from the fast scanning. Figure 10 shows the raster scan patterns with and without using the model-based control. As shown in Figure 11a, the raster scan pattern is distorted if both scan signals are simply standard triangular waves. After using the model-generated, modified triangular waves as shown in Figure 9, the offset and distortion of the raster scan pattern have been successfully corrected (Figure 11b).

**Figure 12:**
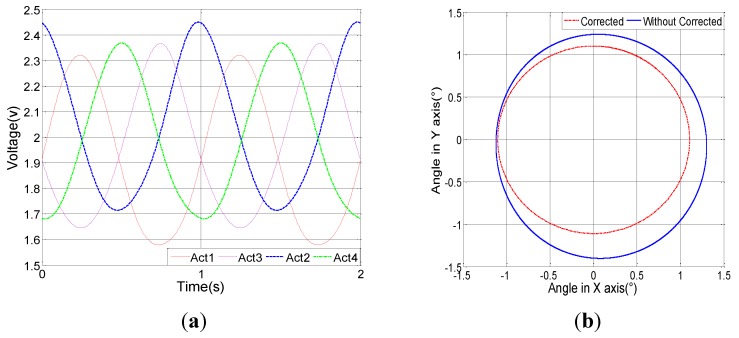
Circular scanning. (**a**) Actuation signals generated by the model-based control system; (**b**) Comparison of corrected and uncorrected circular scan patterns.

If four sinusoidal voltage signals with π/2 phase difference are applied to the four actuators, a circular pattern can be produced. Figure 12a shows the modified sinusoidal voltage signals generated by the model-based control system. As shown in Figure 12b, using the model-generated signals yields an ideal circular scan. On the same figure, a distorted circle is also shown as a comparison, which is the result of standard sinusoidal voltage signals as the input.

### 3.2. Improvement of the Laser Scanning Performance with the Compensated Control System

**Figure 13:**
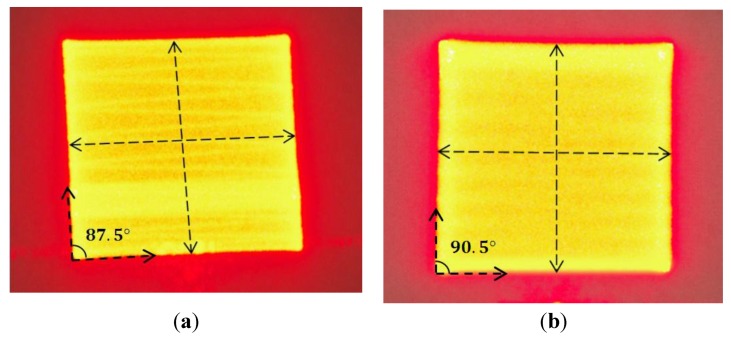
Roster scanning patterns on the screen: (**a**) generated by standard triangle signals; and (**b**) generated by the model-based signals.

Figure 13 shows two raster scan patterns, one obtained by applying standard triangle signals and the other by applying the model-generated signals. Ideally, the contour of the scan area should be a square. However, using just standard triangle signals leads to a slanted square with 2.5° offset (Figure 13a). Changing to the model-generated signals improves to 0.5° offset (Figure 13b).

**Figure 14:**
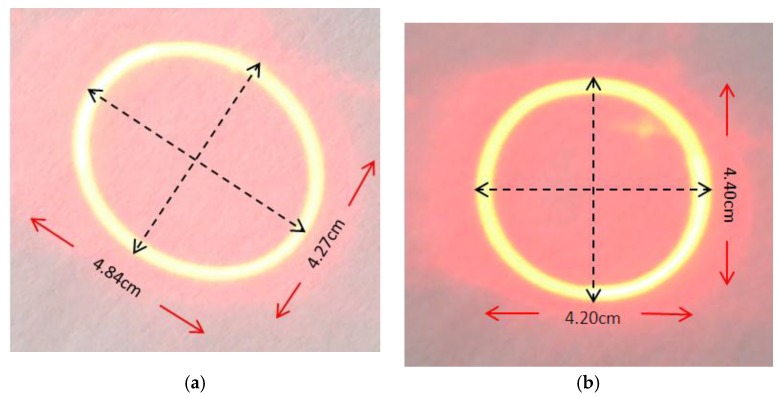
Circular scanning patterns on the screen: (**a**) generated by standard phased-shifted sinusoidal signals; and (**b**) generated by the calculated signals.

Figure 14 shows the experimental results of circular scanning; one was obtained by applying standard 90°-phase shifted sinusoidal signals and the other by applying the model-generated signals. The former pattern is not a circle but more an ellipse; the ratio of the ellipse’s two axes is 1.13 (Figure 14a). The latter pattern is still not a perfect circle, but the ratio of the two axes is improved to 1.05. The eccentricities of the two patterns are 0.471 and 0.298, respectively.

From the above experimental results, we can see that the quality and accuracy of the scan patterns are much improved. However, there are still some small deviations, which are caused mainly by the error of the static model. As the driving voltages increase, the deviation of the model prediction increases. This is because in addition to mechanical coupling there exists thermal coupling among the four actuators. Therefore, a thermal coupling model can be added to the original model to improve the accuracy.

## 4. Conclusions

A mathematical model has been established to predict the mirror plate position of a two-axis MEMS mirror. This four-input, four-output model can predict the full actuation behavior of the MEMS mirror. Experiments show that this model predicts the mirror plate position with only 4.0% maximum error and the average tilt angle error within 0.03°. Based on this model, a simulation system using PI feedback control has been developed to generate corrected driving signals so that the original offsets and deviations can be compensated. Experiments show that this model-based open-loop control can greatly improve the scanning accuracy.
